# Synergistic Antifungal Activity of Chitosan with Fluconazole against *Candida albicans*, *Candida tropicalis*, and Fluconazole-Resistant Strains

**DOI:** 10.3390/molecules25215114

**Published:** 2020-11-03

**Authors:** Wei-Hsuan Lo, Fu-Sheng Deng, Chih-Jung Chang, Ching-Hsuan Lin

**Affiliations:** Department of Biochemical Science and Technology, College of Life Science, National Taiwan University, Taipei 10617, Taiwan; r07b22008@ntu.edu.tw (W.-H.L.); d04b22004@ntu.edu.tw (F.-S.D.); r02b22003@ntu.edu.tw (C.-J.C.)

**Keywords:** *Candida albicans*, *Candida tropicalis*, chitosan, fluconazole, synergistic effect

## Abstract

(1) Background: Few antifungal drugs are currently available, and drug-resistant strains have rapidly emerged. Thus, the aim of this study is to evaluate the effectiveness of the antifungal activity from a combinational treatment of chitosan with a clinical antifungal drug on *Candida albicans* and *Candida tropicalis*. (2) Methods: Minimum inhibitory concentration (MIC) tests, checkerboard assays, and disc assays were employed to determine the inhibitory effect of chitosan with or without other antifungal drugs on *C. albicans* and *C. tropicalis*. (3) Results: Treatment with chitosan in combination with fluconazole showed a great synergistic fungicidal effect against *C. albicans* and *C. tropicalis*, but an indifferent effect on antifungal activity when challenged with chitosan-amphotericin B or chitosan-caspofungin simultaneously. Furthermore, the combination of chitosan and fluconazole was effective against drug-resistant strains. (4) Conclusions: These findings provide strong evidence that chitosan in combination with fluconazole is a promising therapy against two *Candida* species and its drug-resistant strains.

## 1. Introduction

The incidence of fungal infections has increased significantly in recent decades. Current epidemiological surveys have reported that *Candida* species are the leading causes of nosocomial bloodstream infections, which can lead to high mortality rates in at-risk populations [1,2]. However, the issues of currently available antifungal drugs include undesirable side effects and therapeutic failure of the antifungal treatment against drug-resistant strains.

Chitosan, poly-(β-1→4)-2-amino-2-deoxy-D-glucopyranose, is a linear polysaccharide produced by the partial deacetylation of chitin [3,4,5,6,7,8]. Because of its biocompatible, biodegradable, and nontoxic properties, chitosan has been used in many biomedical and therapeutic applications [3,4,5,6,7,8]. For example, halloysite nanotubes coated by chitosan and chitosan nanoparticles have been intensively investigated, and the findings demonstrated that chitosan is a suitable drug delivery system for in vitro and in vivo treatment, thus indicating that chitosan could have promising medicinal applications [9,10,11,12,13]. Additionally, chitosan shows considerable antimicrobial activity against a variety of bacteria and fungi [5,6,7,14,15,16,17,18,19,20,21,22,23,24,25]. However, the mechanisms underlying its antimicrobial activity remain largely unclear. Chitosan has been suggested to exhibit polycationic polymers when the environmental pH is below 6.5 [17,21,23], which leads to interactions with the negatively charged bacterial or fungal cell surface, thereby causing an inhibitory effect [6,7,8,22,23,24].

Combination therapy is generally effective against pathogenic microbes that show drug resistance [26,27,28,29]. The use of chitosan as a fluconazole delivery system or a polymer film containing clinical drugs has been developed to treat infectious candidiasis [30,31]. These studies showed that the advantages of chitosan-based scaffold materials include the control of drug release and the maintenance of a high local concentration of the antibacterial or antifungal drug over a long period of time [30,31]. However, the antifungal efficacy of chitosan in combination with clinical antifungal drugs has been quantified, and the results presented contradictory conclusions [14,32]. Low-molecular-weight chitosan (LMWC; 70 kDa; > 75% deacetylation) exhibited promising anti-*Candida* effects at pH 4.0, whereas the combination of LMWC and fluconazole did not have a synergistic effect at neutral pH [14]. Interestingly, C32, a 15 kDa chitooligosaccharide (CHOS) with 0.15 F_A_ (fraction of acetylation), showed great synergistic effects against *Candida albicans*, *Candida guilliermondii*, and *Candida lusitaniae*, but had an indifferent effect against *Candida tropicalis* when in combination with different antifungals [32]. These data suggest that the molecular weight (MW) and degree of deacetylation of chitosan might result in different outcomes. Nevertheless, previous studies have mainly focused on one chitosan with a low MW or oligo form. Chitosan with different MWs and degrees of deacetylation against *C. albicans* and *C. tropicalis* as well as drug-resistant strains have never been studied.

In this study, six commercial chitosans with distinct MWs and degrees of deacetylation were analyzed to evaluate their antifungal activity and synergistic effects with antifungal drugs against *C. albicans* SC5314 [33], *C. tropicalis* MYA3404 [33], and drug-resistant strains because *C. albicans* and *C. tropicalis* are the most isolated fungal pathogens in tropical and subtropical regions, including Taiwan [34,35,36]. In this study, we first determined the minimum inhibitory concentration (MIC) of chitosan, fluconazole, amphotericin B, and caspofungin. The fractional inhibitory concentration (FIC_index_) determined by checkerboard assays further showed great synergistic antifungal activity against two *Candida* species and the drug-resistant strains in liquid medium after treatment with chitosan-fluconazole, but not chitosan-amphotericin B or chitosan-caspofungin. Together, our findings will reveal new potential and promising therapeutic methods or medical applications to control *Candida* infections.

## 2. Results

### 2.1. Susceptibility of C. albicans SC5314 to Antifungal Drugs and Chitosan with Different MWs

Many review articles have implied that the major mechanism of chitosan against microorganisms is the targeting of the cell wall and cell membrane [4,5,6,7,17,19,22,23,24,31]. Therefore, three classes of antifungal drugs, namely, fluconazole, amphotericin B, and caspofungin, were selected for this study. The in vitro antifungal activities of fluconazole, amphotericin B, caspofungin, and chitosan with different properties were analyzed against *C. albicans* SC5314. As shown in Table 1, the commercial chitosans alone did not exhibit great antifungal activity because the MICs ranged from >2000 μg/mL (3 kDa chitosan oligomer and 20–35 kDa chitosan) to 1000 μg/mL (15 kDa and MMW chitosans), thus corroborating a previous report [32]. Notably, the MIC of HMW chitosan could not be determined because it can only dissolve at higher concentrations of acetic acid (giving a pH < 4.5), and the low pH profoundly inhibited the growth of *C. albicans* (Table 1). Thus, HMW chitosan was not analyzed in the following experiments. Similar to previous reports [37], *C. albicans* SC5314 was highly susceptible to fluconazole (MIC: 0.125 μg/mL), amphotericin B (MIC: 1.0 μg/mL), and caspofungin (0.25 μg/mL).

### 2.2. Chitosan Can Enhance the Antifungal Activity of Fluconazole

**Figure 1:**
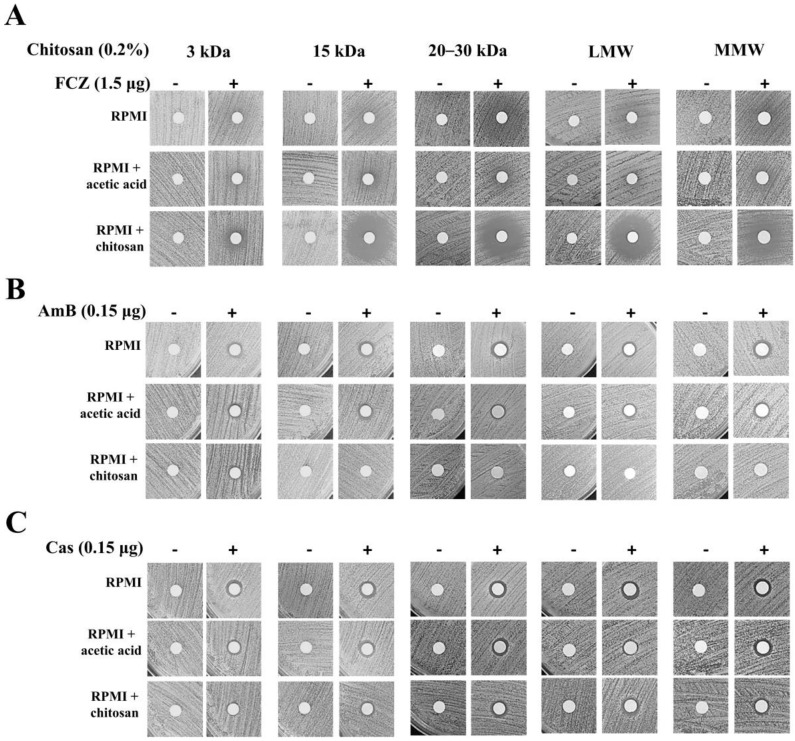
Disk diffusion assay of each chitosan with or without an antifungal drug against *C. albicans* 5314. (**A**) Synergistic effects were observed for chitosan in combination with fluconazole (FCZ) against *C. albicans* SC5314, whereas indifferent effects were observed when chitosan was combined with (**B**) amphotericin B (AmB) and (**C**) caspofungin (Cas).

The susceptibility range of *C. albicans* was evaluated using three antifungal drugs (fluconazole, amphotericin B, and caspofungin) with chitosans presenting different properties (except HMW chitosan). The combination of the abovementioned antifungal drugs and chitosan showed synergistic action with fluconazole and exhibited a remarkable inhibitory effect on *C. albicans* SC5314. The FIC_index_ of each sample in the chitosan-fluconazole checkerboard assay was <0.5 (Table 2). However, chitosan in combination with amphotericin B or caspofungin showed an indifferent effect, with each FIC_index_ between 0.5 and 4.0 (Table 2). To obtain better visualization results, disk diffusion assays were utilized. We further demonstrated that the combined treatment of a particular chitosan with fluconazole exhibited great antifungal activity (Figure 1A); however, obvious inhibitory effects were not observed for the combinations of chitosan-amphotericin B (Figure 1B) and chitosan-caspofungin (Figure 1C). Notably, the 3 kDa and MMW chitosans with fluconazole had no obvious synergistic antifungal effects on *C. albicans* SC5314 in the disk diffusion assays (Figure 1A). Turbid haloes were observed after treatment with fluconazole alone because fluconazole is a fungistatic antifungal drug rather than a fungicidal agent [38]. These data suggest that the synergistic effects against *C. albicans* SC5314 are profoundly affected by the assay that was performed (MIC tests in liquid medium versus disk diffusion assays on agar medium).

### 2.3. C. tropicalis MYA3404 Is Highly Susceptible to Chitosan and Chitosan-Fluconazole Treatment Exhibits Synergistic Effects Against C. tropicalis MYA3404

**Figure 2:**
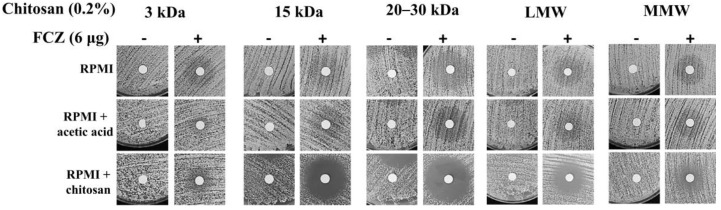
Disk diffusion assay of each chitosan with or without fluconazole against *C. tropicalis* MYA3404. Different MWs of chitosan (except 3 kDa chitosan) with fluconazole exhibited great cytocidal effects on *C. tropicalis* MYA3404.

*C. tropicalis* was selected for this analysis because it is the second most isolated fungal pathogen in Taiwan. The MIC of fluconazole against *C. tropicalis* MYA3404 was 0.5 (Table 3). Interestingly, except for the 3 kDa chitosan oligomer, each chitosan exhibited a remarkable reduction in MIC (ranging from 1.56–7.81 μg/mL) (Table 3) compared with *C. albicans* SC5314 (Table 1). Checkerboard assays of each chitosan with fluconazole (but not with amphotericin B or caspofungin) were performed because of the indifferent effects of chitosan-amphotericin B and chitosan-caspofungin against *C. albicans*, as previously observed (Table 2). Similar to the results obtained for *C. albicans* SC5314, chitosan in combination with fluconazole had a synergistic effect against *C. tropicalis* MYA3404 (FIC_index_ < 0.5) (Table 4). Additionally, the disk diffusion assays also indicated that treatment with chitosan-fluconazole exhibited a larger clear zone than treatment with chitosan or fluconazole alone, although 3 kDa and MMW chitosan did not exhibit a clear inhibition zone in the combination treatment (Figure 2).

### 2.4. Combination of Chitosan and Fluconazole Showed Great Fungicidal Activity Against Drug-Resistant C. albicans and C. tropicalis Isolates

**Figure 3:**
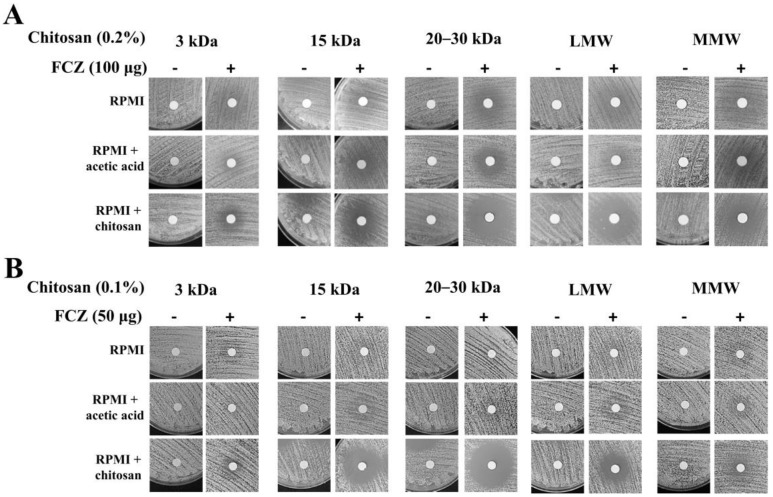
Disk diffusion assay of each chitosan with or without fluconazole against drug-resistant *Candida* strains. Fluconazole-resistant strains of *C. albicans* and *C. tropicalis* were significantly inhibited after treatment the combination of chitosan and fluconazole, particularly 15 kDa, 20–35 kDa, and LMW chitosan.

To further explore whether the chitosan-fluconazole combination treatment is able to inhibit fluconazole-resistant *C. albicans* and *C. tropicalis* isolates, checkerboard assays were performed. First, the MIC values after treatment with fluconazole (8 μg/mL for *C. albicans* and 512 μg/mL for *C. tropicalis* isolates) confirmed that these clinical isolates were fluconazole-resistant strains. Nevertheless, combination treatment exhibited a synergistic antifungal effect in both drug-resistant *C. albicans* (FIC_index_ < 0.5) and drug-resistant *C. tropicalis* (FIC_index_ < 0.5) and showed an excellent inhibition zone after treatment with chitosan with MWs of 15 kDa and 20–35 kDa and LMW (Table 5 and Figure 3).

## 3. Discussion

The influence of the MW and degree of acetylation of chitosan on antimicrobial activity is diverse and has shown different outcomes [39,40,41]. In particular, lower deacetylation degrees and MWs of chitosan (between 42.5 and 135 kDa) exhibited stronger antimicrobial activity against gram-negative bacteria [41]. However, higher MW (300–400 kDa) chitosan and chitosan with a lower degree of deacetylation (but not chitooligosaccharides) had stronger activity against gram-positive bacteria [42]. A similar study indicated that HMW chitosan exhibited better antibacterial activity than chitosan oligomers [39]. These data suggested that the inhibitory effects were also dependent on the pathogen type and chitosan properties as well as the preparation method and chitin source. Indeed, our results showed that the chitosan used in this study exhibited different antifungal effects against *C. albicans* and *C. tropicalis*. The 3 kDa chitosan oligomers and HMW chitosan exhibited lower antifungal effects against *Candida* species. Furthermore, 15 kDa, 20 kDa, and LMW chitosan (but not 3 kDa and MMW chitosan) in combination with fluconazole in the checkerboard and disk diffusion assays showed remarkable antifungal effects.

We observed a hundred- to thousand-fold increase in chitosan susceptibility to *C. tropicalis* compared with *C. albicans*, thus implying that the cell surface properties of the two *Candida* species are different. Previous reports have shown that the contents of unsaturated fatty acids positively influence the membrane fluidity [43]. Moreover, the membrane fluidity of filamentous fungi has been reported to contribute to chitosan susceptibility [44]. Higher membrane fluidity tends to result in a more negative charge on the cell membrane [43,45], thus facilitating cationic chitosan binding to the cell membrane. Previous studies have shown that the cell membrane of *C. tropicalis* contains more polyunsaturated fatty acids than that of *C. albicans*, suggesting that the *C. tropicalis* cell membrane has a greater negative charge than the *C. albicans* cell membrane, although the differences are highly dependent on the analytical methods and culture conditions [46,47,48,49]. Furthermore, glucan, chitin, and mannan carbohydrates and a few proteins constitute the outer layer of the cell walls of *Candida* species [50,51]. Mannoproteins (protein-linked mannan) harboring phosphate groups (phosphorylated mannosyl side chains) also confer a negative charge to the fungal cell wall [50,51,52]. Previous studies have demonstrated that mannan components and the phosphomannan content on the *C. tropicalis* cell wall are significantly higher than those on the *C. albicans* cell wall [53,54], which might explain why *C. tropicalis* exhibited remarkably greater chitosan sensitivity than *C. albicans*.

The mode of action of chitosan against microbes has been investigated and reported in several review articles [3,4,6,7,8,22,24,40]. (1) Chitosan (>50 kDa or higher MW) binds on the microbial cell wall to prevent nutrients from entering the cell, alters the cell permeability, and could act as a metal chelator that inhibits microbial growth [5,6,17,22,55]. Thus, the LMW, MMW, and HMW chitosans used in this study might only target the fungal cell wall. Although the LMW, MMW, or HMW chitosan likely cannot pass through cell wall, because chitosan is a linear polysaccharide, it might be able to penetrate the cell wall and bind to the cell membrane. (2) Chitosan (<50 kDa or lower molecular weight) might also have intracellular activity, thereby affecting the molecular aspects of DNA, RNA, or protein synthesis. Indeed, reports have demonstrated that chitosan (≤50 kDa) and nano-sized particles can penetrate the bacteria cell wall and inhibit DNA transcription [4,56]. Therefore, 3 kDa, 15 kDa, or 20–30 kDa chitosan may not only present antimicrobial activity when interacting with the cell wall and cell membrane, but also present intracellular antifungal effects. Obviously, the molecular size of chitosan determines its antifungal activity intracellularly or extracellularly. The structure rather than the MW of chitosan also plays crucial roles in the location of antifungal activity.

In this study, three chitosans with specific MWs were applied in combination with fluconazole and caused great synergistic antifungal activity against *C. albicans* and *C. tropicalis* as well as drug-resistant strains with liquid and agar media. However, the combinations of chitosan-amphotericin B and chitosan-caspofungin against *C. albicans* showed indifferent effects. Phospholipids and ergosterol (a targeting site of amphotericin B) are important components of the *Candida* cell membrane [57]. The NH_3_^+^ groups of chitosan are believed to be able to bind to negatively charged plasma membranes, thus leading to changes in membrane permeabilization and inhibiting microorganisms [5,6,7,23,38,58]. Amphotericin B consists of mycosamine and macrolactone moieties that can alter the membrane ion permeability and potential on fungal cell membranes [59]. In particular, the mycosamine NH_3_^+^ of amphotericin B is a critical appendage for the binding to ergosterol [59,60,61]. Therefore, competitive inhibition likely occurs between chitosan and amphotericin B on the fungal membrane. However, the mechanisms underlying the indifferent effects of the combination of chitosan with caspofungin remain unclear.

*C. albicans* (~50%) and *C. tropicalis* (~20%) are the most frequently isolated *Candida* species that affect humans in Taiwan [35,62,63]. Moreover, *C. tropicalis* develops fluconazole resistance much more rapidly than *C. albicans* [64]. Indeed, more fluconazole-resistant isolates are observed for *C. tropicalis* (15%) than *C. albicans* (4%) [65]. However, currently available antifungal drugs are limited and ineffective against new and drug-resistant strains [66,67]. Our findings provide strong evidence that chitosan is a promising alternative for combination therapy against *Candida* species and drug-resistant strains. The main issue with this treatment is that chitosan exhibits low antimicrobial activity at neutral pH. Thus, the application of chitosan in combination with fluconazole locally for skin and mucosal infections or a chitosan-based nanoparticle as a fluconazole carrier will likely provide more beneficial effects in clinical therapy. Further in vivo animal experiments to validate the in vitro findings are necessary to provide useful guidelines to develop a better method and formulation to manage fungal pathogens.

## 4. Materials and Methods

### 4.1. Strains and Media

The *C. albicans* and *C. tropicalis* strains used in this study included the sequence SC5314 strain MYA3404 [33] and clinical isolate [68] obtained from National Taiwan University Hospital. YPD, RPMI 1640 (Roswell Park Memorial Institute 1640) and RPMI 1640 supplemented with chitosan were prepared as previously described [69]. The characteristics of each chitosan used in this study are listed below (Table 6). The ~3 kDa chitosan oligomer (>85% deacetylation; cat: OC28900) was obtained from Carbosynth Ltd., United Kingdom. The ~15 kDa chitosan sample (>85% deacetylation; cat: 21161-50) was purchased from Polysciences, Inc., Warrington, PA, USA. Chitosan (20–30 kDa, >90% deacetylation) was purchased from Shin Era Technology, Taiwan (cat: CHG-87G). Low-molecular-weight (LMW) chitosan (50–190 kDa; deacetylation 75–85%; cat: 448869), medium-molecular-weight (MMW) chitosan (deacetylation 75–85%; cat: 448877), and high-molecular-weight (HMW) chitosan (310–375 kDa; deacetylation >75%; cat: 419419) were purchased from Sigma-Aldrich Co., St. Louis, MO, USA. Chitosan must be dissolved in acetic acid before being added to the medium. The final pH of each chitosan-containing medium was 6.2~6.3. Moreover, HMW chitosan required a higher acetic acid concentration for dissolution owing to its low solubility, and the final pH of the HMW chitosan medium was 4.5.

### 4.2. MIC and Checkerboard Assays

The MICs were determined for chitosan, fluconazole, amphotericin B, and caspofungin by broth microdilution [35,70]. The MIC was defined as the concentration of the compound that reduces the turbidity of *C. albicans* and *C. tropicalis* cells by more than 50% (fluconazole) or 90% (chitosan, amphotericin B, and caspofungin) [35,71,72]. A typical synergy checkerboard assay setup was performed using a 96-well plate. Columns 1 to 11 contained twofold serial dilutions of antifungal drug, and rows A to G contained twofold serial dilutions of chitosan. Column 12 contained a serial dilution of antifungal drug alone, and row H contained a serial dilution of chitosan alone.

To test the WT *C. albicans* SC5314 FIC_index_, a checkerboard array synergy experiment was performed in which fluconazole in concentrations of 0.0015625–4 μg/mL was combined with 3 kDa chitosan at concentrations of 62.5–4000 μg/mL, 15 kDa chitosan at concentrations of 62.5–4000 μg/mL, 20–30 kDa chitosan at concentrations of 62.5–4000 μg/mL, LMW chitosan at concentrations of 62.5–4000 μg/mL, or MMW chitosan at concentrations of 15.625–1000 μg/mL. Amphotericin B (0.0015625–4 μg/mL) and caspofungin (0.00390625–1 μg/mL) in combination with each chitosan were tested. To test the FIC_index_ of the *C. albicans* drug-resistance strain, fluconazole in concentrations of 1–256 μg/mL was used and combined with each chitosan at the same concentration range.

To test the FIC_index_ of the WT *C. tropicalis* MYA3404, a checkerboard array synergy experiment was performed in which fluconazole in concentrations of 0.0625–16 μg/mL was combined with 3 kDa chitosan at concentrations of 62.5–4000 μg/mL, 15 kDa chitosan at concentrations of 0.15625–10 μg/mL, 20–30 kDa chitosan at concentrations of 0.3125–20 μg/mL, LMW chitosan at concentrations of 0.15625–10 μg/mL, or MMW chitosan at concentrations of 0.0117188–7.5 μg/mL. To test the FIC_index_ of the *C. tropicalis* drug-resistance strain, fluconazole in concentrations of 4–1024 μg/mL was combined with 3 kDa chitosan at concentrations of 62.5–4000 μg/mL, 15 kDa chitosan at concentrations of 0.9375–60 μg/mL, 20–30 kDa chitosan at concentrations of 0.9375–60 μg/mL, LMW chitosan at concentrations of 0.9375–60 μg/mL, or MMW chitosan at concentrations of 0.15625–10 μg/mL.

An antagonist effect was defined as an FIC_index_ of > 4 [73]. MIC and checkerboard assays were performed with three replicates. The formulas (Equations (1), (2), and (3)) for calculating the FIC indices are listed below:

(1) $$FIC_{A}=\frac{MIC_{A}combination}{MIC_{A}alone}$$

(2) $$FIC_{B}=\frac{MIC_{B}combination}{MIC_{B}alone}$$

(3) $$FIC_{index}=\;FIC_{A}+\;FIC_{B}$$

where *A* represents chitosan and *B* represents fluconazole, amphotericin B, or caspofungin.

### 4.3. Disk Diffusion Assays

For the disk diffusion test, 6 mm disks with different concentrations of fluconazole, amphotericin B, and caspofungin were used according to a previous report with slight modifications [74]. Each disk was placed on the surface of the agar with or without chitosan, the plates were incubated at 37 °C for 24 h, after which images were taken.

## 5. Conclusion

Fungal infections have become a serious issue over the past decade; however, the limited number of antifungal drugs and the rapid emergence of drug resistance strains might lead to incurable fungal infections. Chitosan has been extensively studied for potential applications in biomedical areas. Our investigation demonstrated that the combination of chitosan with a currently available antifungal drug shows a remarkable synergistic antifungal effect. Thus, the innovative application of chitosan should be explored in the future.

## Figures and Tables

**Table 1:** Minimum inhibitory concentrations (MICs) of chitosans with different MWs and deacetylation degrees in combination with antifungal drugs against *C. albicans* SC5314.

Chitosan or Drug	Strain	MIC (μg/mL)
3 kDa chitosan	*C. albicans*	>2000
~15 kDa chitosan	*C. albicans*	1000
20–30 kDa chitosan	*C. albicans*	>2000
LMW chitosan	*C. albicans*	2000
MMW chitosan	*C. albicans*	1000
HMW chitosan:	*C. albicans*	n.d.
Fluconazole	*C. albicans*	0.125
Amphotericin B	*C. albicans*	1
Caspofungin	*C. albicans*	0.25

**Table 2:** Checkerboard assays of each chitosan in combination with an antifungal drug against *C. albicans* SC5314. FIC, fractional inhibitory concentration.

Antifungal Drug	Chitosan	FIC_index_
Fluconazole	3 kDa chitosan	0.5 ± 0.125
	~15 kDa chitosan	0.125 ± 0.035
	20–30 kDa chitosan	0.113 ± 0.038
	LMW chitosan	0.118 ± 0.043
	MMW chitosan	0.041 ± 0.021
Amphotericin B	3 kDa chitosan	1.75 ± 0.25
	~15 kDa chitosan	1.625 ± 0.625
	20–30 kDa chitosan	1.505 ± 0.495
	LMW chitosan	1.05 ± 0.02
	MMW chitosan	1.505 ± 0.495
Caspofungin	3 kDa chitosan	2.515 ± 1.485
	~15 kDa chitosan	1.078 ± 0.048
	20–30 kDa chitosan	2.505 ± 1.495
	LMW chitosan	1.03125 ±0.12
	MMW chitosan	1.578 ± 0.453

**Table 3:** MICs of chitosans of different MWs and degrees of deacetylation in combination with fluconazole against *C. tropicalis* MYA3404.

Chitosan or Drug	Strain	MIC (μg/mL)
3 kDa chitosan	*C. tropicalis*	1000
~15 kDa chitosan	*C. tropicalis*	7.81
20–30 kDa chitosan	*C. tropicalis*	1.56
LMW chitosan	*C. tropicalis*	7.81
MMW chitosan	*C. tropicalis*	7.81
Fluconazole	*C. tropicalis*	0.5

**Table 4:** Checkerboard assays of each chitosan in combination with fluconazole against *C. tropicalis* MYA3404.

Antifungal Drug	Chitosan	FICindex
Fluconazole	3 kDa chitosan	0.375 ± 0.125
	~15 kDa chitosan	0.435 ± 0.165
	20–30 kDa chitosan	0.21 ± 0.07
	LMW chitosan	0.39 ± 0.11
	MMW chitosan	0.42 ± 0.14

**Table 5:** Checkerboard assays of each chitosan in combination with fluconazole against fluconazole-resistant clinical strains.

Drug-Resistant Strain	Chitosan	FICindex
*C. albicans*	3 kDa chitosan	0.375 ± 0.125
	~15 kDa chitosan	0.188 ± 0.063
	20–30 kDa chitosan	0.208 ± 0.168
	LMW chitosan	0.16 ± 0.09
	MMW chitosan	0.085 ± 0.015
*C. tropicalis*	3 kDa chitosan	0.19 ± 0.07
	~15 kDa chitosan	0.26 ± 0.24
	20–30 kDa chitosan	0.265 ± 0.235
	LMW chitosan	0.26 ± 0.24
	MMW chitosan	0.31 ± 0.19

**Table 6:** Chitosans used in this study. LMW, low molecular weight; MMW, medium MW; HMW, high MW.

Chitosan	Molecular Weight	Degree of Deacetylation
Chitosan oligomer	3 kDa	minimum 85%
~15 kDa chitosan	avg. 15 kDa	minimum 85%
20–30 kDa chitosan	20–30 kDa	≥90%
LMW chitosan	50–190 kDa	75–85%
MMW chitosan	Not available	75–85%
HMW chitosan	310–375 kDa	>75%
